# Supplementary material for: Viral protease-mediated polyprotein processing in human astroviruses
Source: Npj Viruses. 2026 Jun 12;4:45. doi: 10.1038/s44298-026-00203-7 (PMC13616911; doi:10.1038/s44298-026-00203-7)
Supplement: Supplementary file 1 [file 44298_2026_203_MOESM1_ESM.pdf]

Supplemental Table 1

Dataset	Peptide	Prev AA	Next AA	Start	End	Peptide Length	Charges	Probability	Spectral Count	Intensity	Assigned Modifications	Protein	Mapped Proteins
Trypsin	ANANKGVTIN	M	V	2	11	10	2,3	1.00	2	7.0E+08	5K(304.2071),N-term(304.2071),N-term(42.0106)	ORF2-MLB2_1	
Trypsin	ANANKGVTINVGN	M	V	2	14	13	2,3	1.00	3	3.5E+08	5K(304.2071),N-term(304.2071),N-term(42.0106)	ORF2-MLB2_1	
Trypsin	ANANKGVTINVGNVKR	M	K	2	17	16	2,3,4	1.00	36	4.0E+10	15K(304.2071),5K(304.2071),N-term(304.2071),N-term(42.0106)	ORF2-MLB2_1	pMLB2_3
Trypsin	QQITADVYR	N	V	348	356	9	2	1.00	2	6.7E+08	1Q(-17.0265),N-term(304.2071)	ORF2-MLB2_1	pMLB2_3
Trypsin	QITADVYR	Q	V	349	356	8	2	1.00	2	2.6E+08	N-term(304.2071)	ORF2-MLB2_1	pMLB2_3
Trypsin	ITADVYR	Q	V	350	356	7	2	0.99	1	3.9E+07	N-term(304.2071)	ORF2-MLB2_1	pMLB2_3
Trypsin	IPDQPIGPSSYPMTPDSLVLMAGYFR	V	A	408	433	26	3	0.99	1	0.0E+00	13M(15.9949),21M(15.9949),N-term(304.2071)	ORF2-MLB2_1	pMLB2_3
Trypsin	DQPIGPSSYPMTPDSLVLMAGYFR	P	A	410	433	24	3	1.00	1	0.0E+00	11M(15.9949),19M(15.9949),N-term(304.2071)	ORF2-MLB2_1	pMLB2_3
Trypsin	QPIGPSSYPMTPDSLVLMAGYFR	D	A	411	433	23	3	1.00	2	5.4E+06	10M(15.9949),18M(15.9949),N-term(304.2071)	ORF2-MLB2_1	pMLB2_3
Trypsin	SYPMTPDSLVLMAGYFR	S	A	417	433	17	3	1.00	2	1.1E+07	12M(15.9949),4M(15.9949),N-term(304.2071)	ORF2-MLB2_1	pMLB2_3
Trypsin	AVDAETKIR	H	R	678	686	9	3	1.00	1	4.2E+07	7K(304.2071),N-term(304.2071)	ORF2-MLB2_1	pMLB2_3
Trypsin	EEGVDAGR	M	S	695	702	8	2	0.83	1	7.2E+06	N-term(304.2071)	ORF2-MLB2_1	pMLB2_3
Trypsin	AVPFHIGVADKVLTLGSSR	M	S	6	24	19	3,4	1.00	3	4.5E+08	11K(304.2071),N-term(304.2071),N-term(42.0106)	pMLB2_3	ORF1a-MLB2_1
Chymotrypsin	ENWQDQTKDY	A	V	173	182	10	2,3	1.00	2	2.0E+09	8K(304.2071),N-term(304.2071)	pMLB2_3	ORF1a-MLB2_1
Chymotrypsin	RVRTGVASF	Q	V	384	392	9	3	1.00	1	1.0E+09	N-term(304.2071)	pMLB2_3	ORF1a-MLB2_1
Trypsin	TNTGYTGGATVIR	Q	A	531	543	13	2	0.98	1	3.4E+07	N-term(304.2071)	pMLB2_3	ORF1a-MLB2_1
Chymotrypsin	SAGPGPKRTSKL	R	R	1309	1320	12	4	0.99	10	1.2E+09	11K(304.2071),7K(304.2071),N-term(304.2071)	pMLB2_3	ORF2-MLB2_1, ORFX-MLB2_3
Chymotrypsin	YGGNGTLGEAIY	Q	Q	1571	1582	12	2	1.00	1	5.0E+07	N-term(304.2071)	pMLB2_3	ORF2-MLB2_1
Chymotrypsin	GNGTLGEAIY	G	Q	1573	1582	10	2	1.00	1	9.3E+07	N-term(304.2071)	pMLB2_3	ORF2-MLB2_1
Chymotrypsin	QQITADVY	N	R	1628	1635	8	2	0.98	2	4.0E+08	N-term(304.2071)	pMLB2_3	ORF2-MLB2_1
Chymotrypsin	SVIPDQPIGPSSYPM	R	T	1686	1700	15	2	0.99	3	4.9E+06	15M(15.9949),N-term(304.2071)	pMLB2_3	ORF2-MLB2_1
Chymotrypsin	IPDQPIGPSSYPM	V	T	1688	1700	13	2	1.00	2	6.8E+07	13M(15.9949),N-term(304.2071)	pMLB2_3	ORF2-MLB2_1
Chymotrypsin	IPDQPIGPSSYPMTPDSL	V	V	1688	1705	18	3	1.00	1	1.6E+07	13M(15.9949),N-term(304.2071)	pMLB2_3	ORF2-MLB2_1
Chymotrypsin	DQPIGPSSYPM	P	T	1690	1700	11	2	1.00	1	2.0E+07	N-term(304.2071)	pMLB2_3	ORF2-MLB2_1
Chymotrypsin	SYPMTPDSL	S	V	1697	1705	9	2	1.00	2	7.0E+07	4M(15.9949),N-term(304.2071)	pMLB2_3	ORF2-MLB2_1
Trypsin	AATGVNQGPVHNLGAEINQSR	M	L	17	37	21	2,3	1.00	17	1.5E+10	N-term(304.2071),N-term(42.0106)	ORF2-pAVIC(capsid)_2	ORF-X-pAVIC_1, pAVIC_3
Trypsin	AHGEPYYSSKPDKDFNFGSTMAR	M	R	30	52	23	3,4	1.00	61	9.8E+09	10K(304.2071),13K(304.2071),21M(15.9949),N-term(304.2071),N-term(42.0106)	pAVIC_2	ORF1a-pAVIC_1, ORF1ab-pAVIC_1
Trypsin	KLKQSGVR	Q	V	444	451	8	4	0.88	1	4.2E+08	1K(304.2071),3K(304.2071),N-term(304.2071)	pAVIC_2	ORF1a-pAVIC_1, ORF1ab-pAVIC_1
Chymotrypsin	SGVRVGVNEF	Q	V	448	457	10	2,3	0.98	3	4.7E+09	N-term(304.2071)	pAVIC_2	ORF1a-pAVIC_1, ORF1ab-pAVIC_1
Trypsin	QKSVSDSDVVDLVR	E	T	656	669	14	3	1.00	1	2.2E+07	2K(304.2071),N-term(304.2071)	pAVIC_2	ORF1a-pAVIC_1, ORF1ab-pAVIC_1
Trypsin	KSVSDSDVVDLVR	Q	T	657	669	13	3	1.00	1	3.6E+08	1K(304.2071),N-term(304.2071)	pAVIC_2	ORF1a-pAVIC_1, ORF1ab-pAVIC_1
Trypsin	AKPIPALR	Q	T	784	791	8	3	0.98	1	8.9E+08	2K(304.2071),N-term(304.2071)	pAVIC_2	ORF1a-pAVIC_1, ORF1ab-pAVIC_1
Trypsin	ASKSNKQVTVEVSNNGR	M	N	1444	1460	17	2,3,4	1.00	104	4.2E+10	3K(304.2071),6K(304.2071),N-term(304.2071),N-term(42.0106)	pAVIC_2	ORF2-pAVIC(capsid)_1
Trypsin	KQVTVEVSNNGR	N	N	1449	1460	12	3	1.00	1	4.0E+07	1K(304.2071),N-term(304.2071)	pAVIC_2	ORF2-pAVIC(capsid)_1
Trypsin	DNASDTLGPSIEIHTLGR	N	T	1655	1672	18	3	1.00	1	3.1E+07	N-term(304.2071)	pAVIC_2	ORF2-pAVIC(capsid)_1
Chymotrypsin	STDNQVPVTF	K	E	1712	1721	10	2	1.00	1	6.3E+07	N-term(304.2071)	pAVIC_2	ORF2-pAVIC(capsid)_1
Chymotrypsin	AGERTTSDTVW	R	Q	1756	1766	11	2	1.00	1	1.2E+09	N-term(304.2071)	pAVIC_2	ORF2-pAVIC(capsid)_1
Trypsin	YVYPSYQDALSNKPALCTGSTPGGMR	F	T	1810	1835	26	4	1.00	2	2.3E+07	13K(304.2071),17C(57.0214),25M(15.9949),N-term(304.2071)	pAVIC_2	ORF2-pAVIC(capsid)_1
Chymotrypsin	TRNPVTTTL	R	Q	1836	1844	9	2	0.99	1	2.0E+08	N-term(304.2071)	pAVIC_2	ORF2-pAVIC(capsid)_1
Trypsin	MNQPSLGHGEAPAAIGR	Q	S	1849	1865	17	3	1.00	1	8.1E+07	N-term(304.2071)	pAVIC_2	ORF2-pAVIC(capsid)_1
Trypsin	NQPSLGHGEAPAAIGR	M	S	1850	1865	16	3	1.00	1	3.6E+06	N-term(304.2071)	pAVIC_2	ORF2-pAVIC(capsid)_1
Trypsin	QPSLGHGEAPAAIGR	N	S	1851	1865	15	3	1.00	1	1.6E+07	N-term(304.2071)	pAVIC_2	ORF2-pAVIC(capsid)_1
Chymotrypsin	SIPAPGEEY	R	K	1866	1874	9	2	1.00	4	1.5E+09	N-term(304.2071)	pAVIC_2	ORF2-pAVIC(capsid)_1
Chymotrypsin	IPAPGEEY	S	K	1867	1874	8	2	0.99	1	2.1E+08	N-term(304.2071)	pAVIC_2	ORF2-pAVIC(capsid)_1

Supplemental Table 2

Raw data for the Figure 5

B, Huh7.5.1

WT				RdRp KO				Pro KO				Q177G			
92.406	90.759	97.896	118.939	0.302	0.314	0.336	0.310	0.389	0.360	0.387	0.424	68.161	90.942	88.838	83.989

Q415G				Q419G				Q415+Q419G				Q628			
4.053	4.501	3.852	3.138	84.995	107.960	102.470	101.555	0.872	0.520	0.462	0.438	36.139	35.865	34.584	30.284

Q664				Q755			
0.861	0.736	0.667	0.763	86.093	91.034	78.774	79.140

B, BSR

WT				RdRp KO				Pro KO				Q177G			
95.865	96.053	98.684	109.398	0.060	0.057	0.054	0.057	0.030	0.039	0.027	0.027	23.120	25.000	24.436	25.940

Q415G				Q419G				Q415+Q419G				Q628			
6.654	7.143	7.876	8.308	56.015	48.120	50.376	58.083	0.034	0.033	0.083	0.047	1.673	1.680	1.788	1.898

Q664				Q755			
0.049	0.066	0.050	0.055	14.586	13.703	14.060	13.120

C, Huh7.5.1

WT				RdRp KO				Pro KO				Q174G			
94.579	112.136	85.805	107.479	0.012	0.007	0.013	0.014	0.022	0.025	0.019	0.017	27.689	21.729	20.485	15.956

Q372G				Q379G				Q372G+Q379G				Q570G			
12.618	13.722	13.055	13.962	2.872	2.867	3.117	2.820	0.050	0.054	0.052	0.040	40.023	36.830	36.457	34.487

Q571G				Q570G+Q571G				Q603G				Q725G			
0.059	N/A	0.064	0.060	0.020	0.027	0.020	0.016	0.135	0.129	0.128	0.144	0.064	0.063	0.067	0.068

C, HEK293T

WT				RdRp KO				Pro KO				Q174G			
89.137	119.106	97.219	94.538	0.240	0.232	0.251	0.216	0.193	0.188	0.186	0.203	59.247	67.550	63.493	66.011

Q372G				Q379G				Q372G+Q379G				Q570G			
13.163	14.133	13.875	16.161	43.419	40.449	46.385	41.538	0.365	0.393	0.317	0.326	120.436	127.962	134.432	124.565

Q571G				Q570G+Q571G				Q603G				Q725G			
0.548	0.684	0.709	0.586	0.316	0.337	0.381	0.284	0.317	0.443	0.324	0.343	0.293	0.282	0.376	0.352

Supplemental Table 3

Raw data for the Figure 6

A

Passage	HAstV1-WT			HAstV1-Q419G			HAstV1-Q628G			HAstV1-Q755G		
0	1.00E+06	1.33E+06	1.47E+06	2.00E+05	3.33E+05	2.67E+05	ND	ND	ND	8.00E+03	9.33E+03	1.33E+04
1	2.33E+07	2.00E+07	1.67E+07	2.20E+07	1.47E+07	2.00E+07	ND	ND	ND	ND	ND	ND
2	1.27E+06	1.27E+06	1.33E+06	9.33E+05	2.07E+06	1.40E+06	ND	ND	ND	ND	ND	ND

B

Time, hpi	HAstV1-WT				HAstV1-Q419G			
0	2.00E+02	3.00E+02	1.00E+02	2.50E+02	1.00E+02	4.00E+02	3.00E+02	2.00E+02
12	1.20E+03	1.10E+03	8.00E+02	5.00E+02	1.40E+03	9.00E+02	7.00E+02	9.00E+02
24	1.10E+06	8.00E+05	7.00E+05	8.00E+05	7.00E+05	8.00E+05	5.00E+05	6.00E+05
36	1.00E+06	7.00E+05	1.20E+06	1.00E+06	1.30E+06	7.00E+05	7.00E+05	9.00E+05
48	4.00E+05	8.00E+05	6.00E+05	2.00E+05	2.00E+05	6.00E+05	6.00E+05	3.00E+05
72	1.00E+06	1.10E+06	6.00E+05	8.00E+05	4.00E+05	2.00E+05	6.00E+05	9.00E+05

C

Passage	MLB2-WT			MLB2-Q372G			MLB2-Q379G			MLB2-Q570G		
0	6.67E+05	1.20E+06	9.33E+05	1.20E+05	1.20E+05	9.33E+04	ND	ND	ND	2.27E+05	3.07E+05	1.73E+05
1	1.40E+06	1.00E+06	1.47E+06	1.00E+05	1.13E+05	1.27E+05	ND	ND	ND	7.33E+05	1.47E+06	1.87E+06
2	4.00E+05	5.33E+05	5.33E+05	5.33E+04	6.67E+04	6.67E+04	ND	ND	ND	6.00E+05	9.33E+05	6.00E+05

D

Time, hpi	MLB2-WT			MLB2-Q570G		
0	ND	ND	ND	ND	ND	ND
24	1.60E+03	1.47E+03	1.60E+03	ND	ND	ND
36	2.80E+04	1.60E+04	1.87E+04	2.40E+03	3.07E+03	2.13E+03
48	1.73E+05	1.60E+05	1.60E+05	2.93E+04	4.27E+04	3.60E+04
60	1.47E+06	9.33E+05	9.33E+05	1.60E+05	1.87E+05	1.60E+05
